# Speed, Sensitivity, and Bistability in Auto-activating Signaling Circuits

**DOI:** 10.1371/journal.pcbi.1002265

**Published:** 2011-11-17

**Authors:** Rutger Hermsen, David W. Erickson, Terence Hwa

**Affiliations:** Center for Theoretical Biological Physics and Department of Physics, University of California at San Diego, La Jolla, California, United States of America; Princeton University, United States of America

## Abstract

Cells employ a myriad of signaling circuits to detect environmental signals and drive specific gene expression responses. A common motif in these circuits is inducible auto-activation: a transcription factor that activates its own transcription upon activation by a ligand or by post-transcriptional modification. Examples range from the two-component signaling systems in bacteria and plants to the genetic circuits of animal viruses such as HIV. We here present a theoretical study of such circuits, based on analytical calculations, numerical computations, and simulation. Our results reveal several surprising characteristics. They show that auto-activation can drastically enhance the sensitivity of the circuit's response to input signals: even without molecular cooperativity, an ultra-sensitive threshold response can be obtained. However, the increased sensitivity comes at a cost: auto-activation tends to severely slow down the speed of induction, a stochastic effect that was strongly underestimated by earlier deterministic models. This slow-induction effect again requires no molecular cooperativity and is intimately related to the bimodality recently observed in non-cooperative auto-activation circuits. These phenomena pose strong constraints on the use of auto-activation in signaling networks. To achieve both a high sensitivity and a rapid induction, an inducible auto-activation circuit is predicted to acquire low cooperativity and low fold-induction. Examples from *Escherichia coli's* two-component signaling systems support these predictions.

## Author Summary

Different times call for different measures. Therefore, cells adjust their protein levels depending on their environment. Upon the detection of certain environmental signals, transcription factors are activated, which activate or inhibit the production of specific sets of proteins. As it turns out, these transcription factors often also stimulate their own production. Indeed, such self-regulation is a common motif in signal–response systems of many organisms, including bacteria, animals, plants and viruses–but its function is not well understood. We have used mathematical models to study its benefits and drawbacks. On the one hand, calculations show that self-regulation can be a very useful tool if the cell needs to respond in a sensitive way to changes in its environment, or if it is supposed to respond only if the signal exceeds a threshold level. On the other hand, these benefits come at a cost: self-regulation severely slows down the cell's response to changes in the environment. We have analyzed how the cell can benefit from the advantages of self-regulation, while mitigating the drawbacks. This leads to strict design constraints that examples from the bacterium *E. coli* indeed seem to obey.

## Introduction

Biological organisms employ a variety of signaling networks to respond to changes in environmental conditions. An interesting class of examples is given by the two-component signaling (TCS) systems, which are ubiquitous in bacteria and plants [1]. TCS systems typically consist of two proteins: a sensor histidine kinase (HK) and a response regulator (RR). The HK is a transmembrane protein that auto-phosphorylates in response to a “signal”. The phosphate group of the HK is subsequently transferred to the RR, which in its phosphorylated form usually acts as a transcription factor. As a result, the RR activates its target genes only when the signal is present. TCS systems are the predominant signaling motifs in bacteria; *E. coli*, for instance, features about 30 TCS systems [1]. Interestingly, in about half of the cases, the RR also activates its own expression. The functions of this positive feedback are not well understood [2], [3].

**Figure 1:**
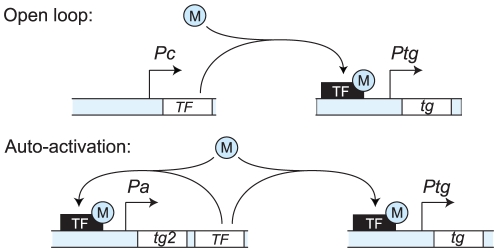
Open-loop vs. auto-regulation circuits. We consider genetic circuits consisting of a transcription factor (TF) that can regulate its target gene, gene *tg*, only if it is activated in response to some signal (shown here as modification M). We compare two alternative designs. In the first design, the open-loop circuit, the TF is constitutively expressed from promoter *Pc* and regulates its target gene by binding to its promoter, *Ptg*. In the second design, the auto-activation circuit, the TF in addition activates its own expression, from promoter *Pa*. In this case, additional genes in TF's operon–such as gene *tg2*–also respond to the signal. Both circuits are ubiquitous in nature.

Fig. 1 illustrates the transcriptional circuit of TCS systems. It consists of a transcription factor (the RR) that has to be modified post-transcriptionally in order to regulate its target genes; in addition, it may activate its own transcription. Gene networks of this type do not only occur in TCS systems, but are in fact a common motif in many organisms, including eubacteria, archaea, eukaryotes, and viruses [2], [4]. While in TCS systems the RR is modified by phosphorylation, many other transcription factors (TFs) are activated by other covalent modifications or by the binding of a ligand. Here, we use mathematical models to study the characteristics of such inducible auto-activation circuits.

Intuitively, the auto-activation and open-loop circuits each possess their distinct advantages [2], [5], [6]. In the open-loop circuit, the TF is expressed constitutively. As a result, the circuit can be induced quickly, because the post-transcriptional processes that activate the TF are rapid, typically occurring in seconds or less. In contrast, the full induction of the auto-activation circuit involves transcription and translation of the TF, which takes minutes [7]. On the other hand, in the open-loop circuit, the TF is produced even if the signal is absent for a long time. The constitutive presence of numerous TFs in high copy numbers could lead to cross-talk or noise, for instance due to spontaneous phosphorylation. These problems are alleviated in the auto-activation circuit, in which the TF level is reduced in the absence of the signal. In addition, positive feedback is generally expected to increase the sensitivity of the response. However, auto-activation can also lead to bistability and hysteresis [8]. While in some circuits bistability can perhaps be beneficial, in signaling circuits that are supposed to provide a well-defined output to a given input level, bistability and strong hysteresis should presumably be avoided. The significance of each of these effects clearly depends on the parameters of the circuits. Below, we examine the above effects using quantitative models and determine which parameter range could combine the benefits of auto-activation while minimizing its drawbacks.

Our results show several surprises. First of all, they demonstrate that an inducible auto-activation circuit can generate an ultra-sensitive threshold response, even if the activation mechanism is non-cooperative. This is surprising, because in open-loop systems sensitivity is associated with molecular cooperativity, either through the cooperative binding of TFs to multiple binding sites at the promoter, or by cooperativity in the activation of TFs. These new results emphasize that auto-activation is an excellent tool for signaling circuits that require a threshold or switch-like response. However, this benefit comes at a cost: stochastic models reveal that the induction speed is strongly affected by auto-activation–in fact, much more so than previously estimated based on deterministic rate equations [2], [3], [7], [9]. The discrepancy between deterministic and stochastic models is most pronounced when the basal transcription rate of the TF is low, in which case rate equations dramatically underestimate the induction time. Moreover, in this regime the induction time also becomes very unpredictable. We show that these effects are expected to occur under conditions that are fairly typical for bacterial circuits. These novel findings demonstrate that the need for a rapid and reliable induction severely constrains the use of auto-activation in response circuits.

Below, we first introduce the model used in this study. Next, we discuss results regarding bistability, sensitivity, and induction speed. We finally combine these results to explore how these characteristics may restrict the designs actually found in nature.

## Models

We consider the inducible circuits illustrated in Fig. 1, consisting of a TF that must be activated to function and possibly activates the transcription of its own gene. To keep the analysis general, we do not specify the nature of the modification, nor the environmental signal triggering the TF's activation. Instead, we assume that in steady state, at a given signal level, a fraction 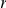 of the TFs will be activated. Thus, 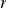 can be considered the input of the circuit. If 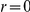, the signal is completely absent, while if 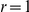 the signal is saturating.

We use a simple, deterministic model to derive our first results [10]. The dynamics of the TF concentration 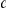 are described by the following ordinary differential equation:
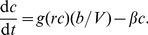
(1)Here 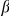 is the degradation rate constant of the TF; in growing cells, 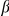 also accounts for dilution due to growth. The TF's transcription rate 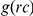 is a function of the concentration of modified TFs, 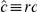, because only the modified TFs can activate transcription. We assume 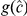 has the following Hill-type form:
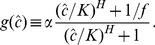
(2)Parameter 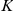 is the dissociation constant of the modified TF binding to its operators, and 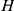 is the Hill coefficient. In this notation, the basal transcription rate is 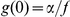, and the maximal transcription rate at full activation is 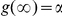, showing that 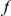 is the maximal fold change of the promoter. If 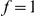, the auto-regulation is eliminated and the model describes the open-loop circuit. Lastly, we assume that each mRNA transcribed from the promoter is instantly translated 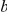 times (the “burst size”) [11]. This results in an increase in the TF concentration by an amount 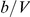, where 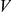 is the volume of the cell. Note that for simplicity we do not explicitly include the dynamics of the mRNAs and we neglect time delays due to transcription, translation and protein folding.

For a given input 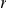, the dynamics of Eq. 1 define a steady state TF concentration 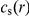 that is at most 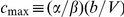. The function 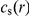 therefore describes the response of the total TF concentration to the signal 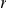. The expression level of genes encoded in the same operon as the TF, such as *tg2* in Fig. 1, is expected to be proportional to 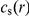, too.

Another important quantity is the steady state concentration of *modified* TF, 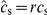. Because only the modified TF regulates the target genes, we consider 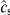 to be the output of the circuit; we will call 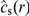 the response function of the circuit. The *shape* of the response function is determined only by parameters 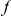 and 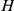 (see Supporting Text S1). We therefore focus on the role of these parameters.

For our analysis of the induction speed, a stochastic version of the above model is required; it will be introduced below, in the section “Achieving rapid induction”.

## Results

### Avoiding bistability and hysteresis

It is well known that auto-activation can lead to bistability [4], [12]–[15]. When an auto-activating circuit is bistable, it has two stable steady states: one in which the TF concentration is high and stays high because the TF activates the transcription of its own gene, and one in which the TF concentration is low and stays low because the TF's gene is not activated.

**Figure 2:**
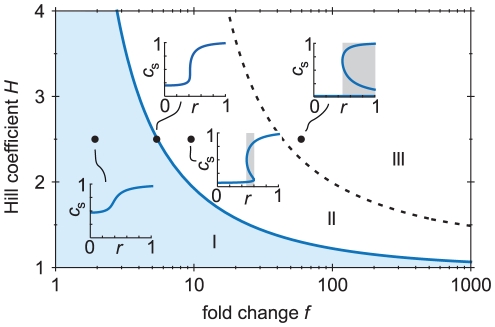
Phase diagram showing which parameter values lead to bistability. When the fold change and the Hill coefficient are both low (regime I), the response of the TF concentration is not bistable (see inset). Above the blue, solid line, however, bistability sets in for a range of input values 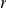. Above the dotted line (regime III), the bistability remains even when the signal is saturated (*i.e.*, if 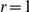). Response circuits for which bistability and hysteresis are not desired, are restricted to the shaded parameter regime. (The blue line does not depend on other parameters; the dotted line depends on the ratio 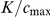, here chosen to be 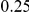.)

Fig. 2 summarizes which parameter values yield bistability in our model. As long as the fold change 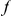 and the Hill coefficient 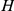 of auto-activation are low, bistability cannot occur (regime I in the diagram). More precisely, bistability does not occur as long as (see Supporting Text S1)
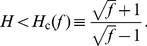
(3)(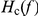 is the solid, blue line in Fig. 2.) In this regime, for any input 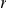, the circuit has a unique steady-state TF level 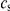 (see the insets in Fig. 2). When 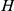 exceeds the critical value 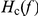, bistability sets in, but only for an intermediate range of 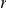 values. For high values of 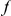 and 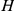, this regime extends all the way up to 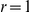 (regime III, to the right of the dotted line). In this regime, the circuit is still bistable even when the signal is saturating.

Regime III seems inappropriate for a signaling circuit. Even at saturating signal levels (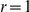), the circuit will not be induced, because the low-expression state remains stable. (In stochastic models the system will eventually turn on by a random fluctuation, but the induction will be very slow, as demonstrated below.) In regime II the circuit is bistable for a range of 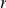 values. There, the output is not uniquely determined by the input, because two output levels are compatible with a given 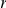. This behavior leads to hysteresis [16]. Bistability and hysteresis can presumably be beneficial in some systems [2], in particular if the circuit has to be cautious in turning on or off, or in the context of bet hedging [17]. However, in signaling systems, assuming that there is an optimal expression level for any given signal level, bistability and hysteresis will tend to trap the circuits in a non-optimal state. We argue therefore that a bistable response is often not desired. Based on these considerations, we expect that the circuit parameters should usually be in regime I (also see the discussion in Ref. [18]).

### Achieving sensitivity

An advantage of positive feedback is that it can increase the sensitivity of a circuit [5], [6]. In the context of dose-response curves, a high sensitivity can be beneficial. In particular, highly sensitive signaling circuits allow the cell to ignore low-level signals, below a certain threshold value, which may be due to noise or cross-talk. We here quantify how much the sensitivity of signal-response circuits can benefit from auto-activation.

The sensitivity of response curves can be defined in various ways [19]. A common approach is to define the sensitivity of a response function 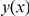 as the maximum slope of that function in a log–log plot, that is, as 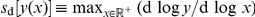. This definition has many desirable properties: a high 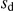 indeed indicates that 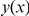 increases rapidly in some domain, the measure is invariant under scaling (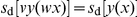), and convenient for mathematical analysis. For those reasons, we will use this measure below. However, a pitfall is that, in order for 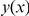 to be deemed sensitive, a high log–log slope in a single point is sufficient. As a result, a high 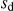 does not guarantee that the circuit behavior resembles a binary switch [19]. Therefore, we also report results for the measure 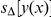, defined as the *average* slope of 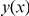 in a log–log plot, calculated over the domain in which it switches from low to high. (In other words: 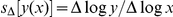 in the switching domain.) The switching domain is defined heuristically as the domain in which 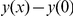 increases from 10% to 90% of its maximum value.

Ultimately, the most relevant quantity is the sensitivity with which the expression of the target genes responds to changes in the signal level. This sensitivity is shaped by each step in the response network: the detection of the signal, the signal transduction, the modification of the TF, and the promoters of the target genes. Consequently, a sensitive response can be implemented at different places in the response network. Here we study the sensitivity that is contributed by the auto-activation circuit and therefore focus on the sensitivity of 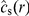.

That auto-activation can strongly improve the sensitivity can be understood by revisiting Fig. 2. If the parameters are chosen near the border between regions I and II, the response is *almost* bistable, leading to a high log–log slope (see inset in Fig. 2). Indeed, for the model in Eq. 1 and 2, 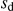 can be calculated exactly (see derivation in Supporting Text S1):
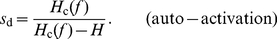
(4)(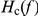 was defined in Eq. 3.) This confirms that the maximal log–log derivative diverges when 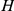 approaches the critical value 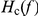. We conclude that an arbitrarily high 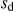 can be obtained for *any* Hill coefficient by properly choosing the fold change, and *vice versa*.

**Figure 3:**
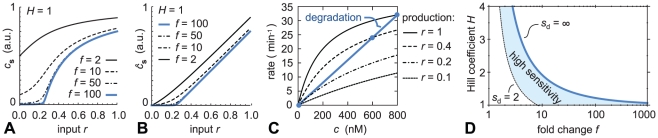
Auto-activation, even if non-cooperative, can strongly increase the sensitivity of the circuit. Fig. A: TF concentration 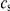 as a function of 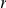, for the non-cooperative auto-activation circuit (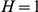). For large values of the fold-change 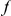 the plot becomes a highly effective threshold response, with threshold 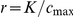, here set to 0.25. Fig. B: Same as Fig. A, but now showing the response of the active species, 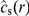, which at large 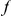 becomes threshold-linear. Fig. C: Graphical explanation of the threshold response at large 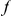. Plotted are both the degradation and the production rate of the TF, as a function of the TF concentration 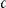 (see Eq. 1). The intersections between these curves (indicated with dots) indicate the steady state level 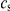. When 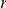 is high, the steady state expression level of 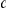 is high. However, when 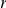 is reduced below 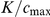 the expression is fully turned off. Fig. D: Phase diagram of the sensitivity, defined as the maximum slope in a log–log plot 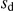 (similar results are obtained using an alternative definition 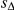; see Supplementary Text S1). Above the solid, blue line the system is bistable (see Fig. 1); on this line 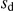 diverges. To obtain a high sensitivity, one therefore has to choose the parameters close to this line, in the shaded region (we use the arbitrary cutoff 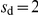).

In particular, if the auto-activation is non-cooperative (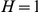), Eq. 4 reduces to
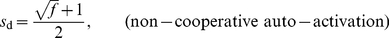
(5)proving that, even in the absence of molecular cooperativity, 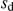 increases without bound when 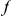 is increased. Indeed, in the limit of large 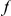 a strict threshold response is obtained. We illustrate this in Fig. 3A and B. Assuming 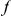 is large, the expression of the TF, 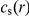, is fully inhibited when 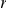 is below the threshold 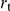; for 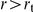 it takes the form of a translated (shifted) Michaelis–Menten curve (Fig. 3A). In the same limit of high 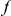, the concentration of activated TF 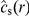 is threshold-linear (Fig. 3B). (Mathematical derivations are provided in the Supplementary Text S1.)

Fig. 3C graphically explains the origin of this behavior. It plots the rate of production of TFs, 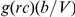, as a function of the TF concentration, for four signal levels; the degradation rate 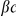 is shown too. The steady state value of 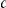 is the value at which production and degradation balance, *i.e.*, where the production and degradation lines cross (indicated with dots). If 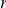 is large, the steady state level is large. But if 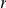 is reduced enough, the production line shifts below the degradation line and the steady state becomes 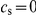. The threshold value is 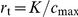 and can be varied biochemically by tuning 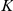.

To illustrate how remarkable the values of 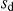 are that can be achieved using auto-activation, we reexamine the open-loop case (see Fig. 1). The open-loop circuit itself is insensitive (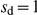), but this can be compensated by choosing a sensitive promoter for the target genes [20]. This requires that the TF binds cooperatively to multiple binding sites on the target promoters. If the TF binds fully cooperatively to 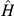 binding sites and achieves a fold change 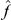 (again assuming the Hill form of Eq. 2), the expression of the target gene responds with the following sensitivity to changes in 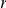:
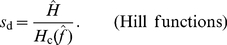
(6)(We provide a derivation in Supplementary Text S1.) Because 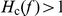, this shows that in open-loop circuits the sensitivity 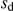 at best equals the number of cooperative binding sites at the promoter. However, this maximal 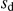 is obtained only if the fold change is large, so that 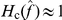. The following numerical example illustrates this point. If the fold change in the non-cooperative auto-activation circuit is 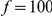, the resulting 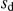 is 5.5. To obtain the same value in the open-loop circuit, at 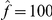, more than 7 cooperative TF binding sites are required at the target promoter. Clearly, if a threshold response is desired, auto-activation can be an excellent tool.

We repeat, however, that even though 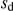 for the non-cooperative auto-activation circuit diverges in the limit of large 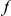, the response function does not converge to a step function (as Hill functions do in the limit of high 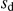), but to the threshold-linear response in Fig. 3B. While this is obviously an excellent threshold response, its quality as a switch is better represented by the measure 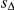. Unlike 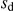, 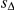 does not diverge in the large-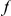 limit, but it nevertheless acquires large values. For instance, in the example above (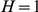, 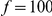, and 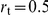), we find 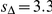. For comparison, for Hill functions,
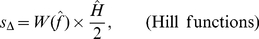
(7)where 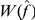 converges to 1 from below as the fold change 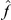 increases (see derivation in Supplementary Text S1). From this it follows that, to obtain the value 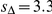 in the open-loop circuit (again assuming 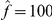), the promoter of the target gene should have a Hill coefficient 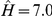 . This shows that non-cooperative auto-activation circuits can constitute an excellent switch.

Fig. 3D summarizes the regions of parameter space that lead to a high sensitivity. The figure is based on Eq. 4 for 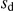. The line 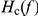 where 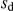 diverges is shown–the same line that marks the boundary between mono- and bistable regimes in Fig. 2. If the circuit is to be sensitive, the parameters have to be close to that line, within the shaded region (where we chose a somewhat arbitrary cutoff 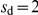). An analogous figure based on 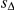 is presented in the Supplementary Text S1 and leads to very similar conclusions.

### Achieving rapid induction

Another important characteristic of a signaling circuit is its induction speed. A circuit that can be induced rapidly in a changing environment is expected to have a fitness advantage [7], [9]. We therefore study the induction time of the circuit.

As we explain below, the deterministic model is not adequate to describe the induction time of the circuit. We therefore introduce a stochastic model. This model is based on the following Master equation, describing the evolution of the probability distribution 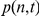 for the TF copy number 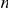 at time 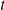:
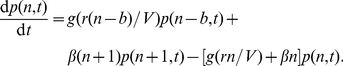
(8)All parameters are analogous to the deterministic model. As in the deterministic case, the mRNA level is not included explicitly but TFs are produced in bursts of size 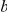. We do not model the binding and unbinding of the TF to the DNA explicitly, but assume that the binding kinetics of the TF at the promoter are fast compared to the time scale of transcription initiation [21]. These assumptions simplify the analysis but are not critical: similar results can be obtained with other models previously presented in the literature [21]–[27].

To introduce the induction time, we imagine that the signal has been absent (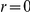) for a period long enough to ensure that the circuit is in steady state. Then, at time 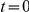, the signal is introduced at a saturating level (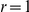). We then define the induction time as the waiting time before the expression of the TF arrives at 50% of its steady state level. In the deterministic model, this can be calculated by solving the differential equation 1. In the stochastic model, the waiting time is actually a random variable; therefore, we report the *mean* waiting time, which can be calculated exactly from the Master equation 8. In the Supporting Text S1 we describe the method used; there we also discuss the full induction time probability distributions.

**Figure 4:**
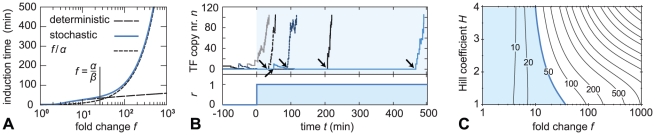
Auto-activation strongly affects the time required for induction. Fig. A: The induction time (defined in the main text) as a function of fold change 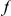, for the case 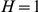 (no cooperativity). In the deterministic model, the induction time depends only mildly on 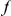. However, at large 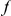 the stochastic model deviates strongly from the deterministic one and predicts a dramatic slowdown. The slowdown sets in when the steady state distribution becomes bimodal, that is, when 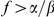; then, the induction becomes limited by the first transcription events, occurring at approximately the basal rate 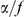. Fig. B: Typical time traces for the induction process, obtained by kinetic Monte Carlo simulations, at 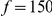. The waiting time for the first transcription event (indicated with arrows) is indeed rate-limiting. Fig. C: Contour plot of the induction time in the stochastic model (in minutes). At 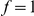 the auto-activation is removed and the induction is instantaneous. If we arbitrarily require that the response time is at most 50 minutes, only the shaded region is adequate. Parameters: 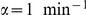, 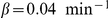, 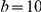, 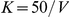.

Fig. 4A shows results from both the deterministic and the stochastic model. Plotted is the induction time as a function of the fold change. We assume that the maximal expression level of the circuit is prescribed by the functional context of the circuit; therefore, we vary 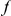 but keep 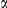 fixed. The deterministic model predicts that the induction time increases mildly–less than two-fold–as 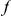 is increased from 10 to 1000. Based on such results, one might conclude that the effect of auto-activation on the induction time is mild. The stochastic model, however, reveals a different picture. When 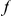 is low, both models are in agreement. But when 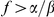 (as we explain below), the stochastic model deviates dramatically from the trend predicted by the deterministic theory, demonstrating that the induction time is much more strongly affected by the auto-activation than expected from deterministic rate equations [2], [3], [7], [9].

What causes the discrepancy between the deterministic model and its stochastic counterpart? When the fold change is increased while the maximal transcription rate 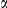 is kept fixed, the basal transcription rate 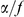 is reduced. As a result, the expected number of TFs present in the cell at time 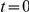
–just before the signal arrives–becomes small. This means that, when the signal is introduced, the TF concentration is initially too low to activate the TF's transcription significantly, so that the transcription rate remains of the order 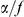. Crucially, this means that the expected waiting time before the first transcription event occurs is close to 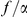. Using realistic parameters for bacteria, in which the maximal transcription rate 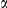 is of the order 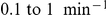, this waiting time can easily become large. Indeed, Fig. 4A shows that for large 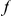 the induction time is of order 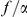, indicating that the first transcription events become the limiting step of the induction. This effect is not accounted for by the deterministic model, which disregards the discreteness of the molecular events.

To visualize the process, Fig. 4B shows five representative time traces of the induction, obtained by kinetic Monte Carlo simulations, for a circuit with a large fold change 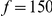. Before the signal is switched on, the copy number fluctuates around the average value 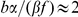. The signal is introduced at time 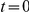. The traces clearly show that the induction time is dominated by the waiting time before the first transcription event; next, the circuit usually switches on rapidly. This has another important consequence: because transcription is (modeled as) a Poisson process, the probability distribution of induction times is approximately exponential (see Supplementary Text S1). The standard deviation of the induction times is therefore as large as the mean. This means that, in this parameter regime, the induction process is not only slow, but also unpredictable.

The anomalous induction time ultimately results from a low basal transcription rate 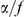. However, assuming that the maximal expression level of the TF is set by its biological function, so that 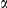 can be considered given, this directly leads to constraints on 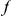. Fig. 4C is a contour plot of the induction time according to the stochastic model. It clearly shows that large fold changes lead to long induction times. Also, unless the fold change is small, increasing the Hill coefficient strongly slows down induction; comparison to Fig. 2 shows that this is because the circuit then approaches and eventually enters the (deterministically) bistable regime. If we arbitrarily decide that the induction time should not exceed 50 min, the admissible values of 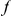 and 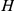 are limited to the small shaded region.

### Relation between slow induction and bimodality

To be precise, we distinguish between bistability and bimodality. We call a circuit bistable if the deterministic model predicts two stable steady states, and bimodal if the stochastic model predicts a steady state probability distribution with two peaks. Naively, one might expect that, in order for a circuit to be bimodal, it should be bistable. Theoretical work has shown, however, that this correspondence does not always hold [24], [26], [28], [29]. In particular, stochastic models of auto-activation circuits can produce bimodality even if the auto-regulation is non-cooperative (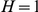), in which case the circuit cannot be bistable (see Fig. 2) [24], [26]. Recently, this theoretical result was verified experimentally using a synthetic auto-activation circuit in *Saccharomyces cerevisiae* [27]. As we will explain, this phenomenon is directly related to the slow induction time we just described.

We explained that the slow induction was due to the fact that, if the circuit is prepared in a state with no TFs (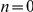), it on average lingers in that state for a time 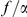, which becomes large if 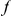 is large. Obviously, the same lingering time holds if the circuit arrives in a state with 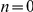 by a rare random fluctuation. Therefore, as 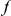 is increased, the stochastic circuit will spend an increasing fraction of its time in the 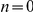 state, which for large enough 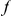 results in a peak in the steady-state distribution at 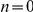. (In fact, in the limit 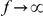 the state 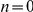 becomes an absorbing state, so that the steady-state probability distribution becomes concentrated entirely on 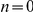.) If another peak is present at non-zero expression levels, a bimodal distribution results. In other words, the anomalous bimodality and the slow induction are different manifestations of the same underlying characteristics of the circuit.

It can readily be derived from the Master equation 8 that the bimodality for non-cooperative auto-activating circuits requires that 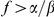 (see Supporting Text S1). In other words, in the non-cooperative circuit, bimodality is obtained only if the basal transcription rate 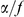 is smaller than the protein degradation/dilution rate 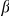. This holds independent of 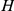, 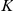 or 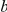; moreover, more detailed stochastic models yield the same result (see supplementary text of Ref. [27]). Given the relation between the anomalous induction time and bimodality, this explains why the induction time of the stochastic model deviates noticeably from the deterministic one when 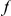 ≳ α/β, as we indeed observed in Fig. 4A.

The above interpretation also indicates that a large burst size 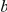 is not required to obtain bimodality at 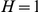 [27]. Bimodality can occur at any burst size, provided the fold change is large enough, such that 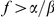 (or, equivalently, provided the basal expression level is low enough such that 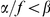). Yet, the burst size can be important in an indirect way: to maintain a fixed steady state expression level, an increased burst size has to be compensated by a decreased 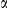 or an increased 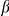. In both cases the requirement 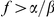 is relaxed.

In models that explicitly treat the binding of the TF to its own promoter, bimodality can also occur due to a different mechanism [21], [25]. If the binding kinetics of the TF are slow, the steady state distribution can have two peaks: one corresponding to the expression when a TF is bound to the promoter, and one corresponding to the expression when the promoter is unbound. For this type of bimodality the duality with an anomalous induction does not necessarily hold.

### Synthesis

**Figure 5:**
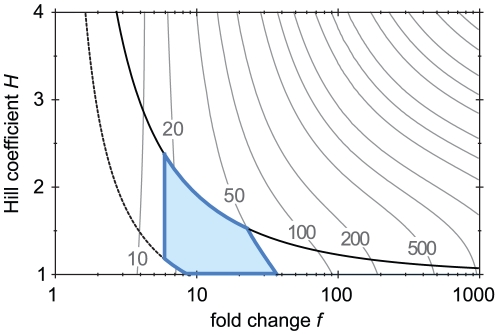
Summary of results. Only in the shaded area is the circuit mono-stable, sensitive (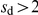), reasonably fast (induction time 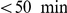), with a non-negligible fold-change (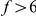). Of course, the precise borders of the area depend on the stringency of the circuit's functional requirements. Based on these results, inducible auto-activation circuits should have an at most moderate fold change; also, a modest Hill coefficient between 1 and 2 is sufficient. This can be achieved by auto-activation mediated by a single TF dimer.

We have assumed that signaling circuits generally care about speed and sensitivity, and should avoid bistability and hysteresis. Each of these properties imposes constraints on the auto-regulation by the TF, as shown in the phase diagrams Figs. 2, 3D and 4C. In Fig. 5 we combine these results to analyze which parameters are compatible with all these constraints. To eliminate bistability, the operating point of the circuit should be below the black solid line. To obtain sensitivity, it should be close to this line. To avoid a slow induction, the fold change should not be too large; yet, to have any benefit from the auto-activation, it should not be too small. These constraints restrict the system to the small parameter region indicated in the plot. In this region, the fold change is at most moderate (≲ 40), and the Hill coefficient is roughly in the range 1 to 2. Such a low Hill coefficient can be achieved using auto-regulation by a single TF dimer.

## Discussion

We analyzed the properties of inducible auto-activation circuits to find parameter regions that are compatible with the requirements of signalling systems. For that reason, we studied bistability, sensitivity and induction speed, and discovered several new phenomena.

First of all, we found that auto-activation circuits can create an ultra-sensitive threshold response, even in the absence of molecular cooperativity. This conclusion holds for both definitions of sensitivity that we studied. To achieve similar levels of sensitivity in open-loop circuits, the promoters of all target genes have to be very sensitive, requiring many cooperative TF binding sites. As this is not feasible for every TF, positive feedback seems an excellent way to greatly increase the sensitivity of the response of *all* target promoters through the construction of a *single* binding site.

However, auto-regulation comes at a cost. We demonstrated that the induction time is much more severely affected by auto-activation than previously appreciated. When the basal transcription rate of the TFs promoter is small, ordinary rate equations fail and stochastic models should be used. In this regime, the waiting time until the first transcription event takes place becomes rate limiting, which makes the induction both slow and unpredictable. This effect imposes strong constraints on the use of auto-regulation for signaling. As a rule of thumb, the basal transcription rate 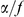 should be such that the average waiting time for the first transcription event, 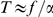, is safely below the required induction time. To indicate the severity of this limitation we note that if 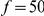 and 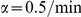, the response time will be more than 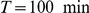. We therefore expect that this new stochastic effect will be relevant under fairly typical bacterial conditions.

Together, the constraints imposed by speed, sensitivity and bistability restrict the parameters to the small shaded area delineated in Fig. 5. Of course, the exact position of the borders of this area depend on somewhat arbitrary choices. If a narrow domain of bistability can be tolerated, somewhat higher Hill coefficients can be admitted. The induction time required in real circuits presumably varies, and therefore the restrictions on the fold change will vary too. Nevertheless, Fig. 5 clearly illustrates the trade-offs that may shape the parameters of response circuits.

For simplicity, we have discussed the sensitivity and the bistability of the circuit in terms of the deterministic model. However, similar results can be obtained in the framework of the stochastic model. In the Supporting Text S1, we demonstrate that the threshold response obtained in the deterministic model is preserved in the stochastic one. There, we also show that the bistable region of parameter space is virtually identical to the region that produces bimodality in the stochastic model–that is, apart from the anomalous bimodality at 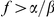 that we discussed above. (How the *stability* of the deterministic steady states is affected by noise has been discussed in detail in Ref. [10].)

With these ideas in mind, we revisit the TCS systems of *E. coli*, which we discussed briefly in the introduction. In order to model any *specific* TCS system quantitatively, the general models that we presented have to be extended to include the complex details of the signal transduction and the expression of the sensor kinase [3], [18], [30]. Nevertheless, the constraints that we derived should also affect TCS systems. To our knowledge, the *E. coli* genome contains 26 RRs, 14 of which are believed to auto-regulate. Table 1 lists these auto-regulating RRs. From this table, it is clear that a significant fraction of RRs *activate* their own expression level. Table 1 also mentions the number of binding sites found for the RR at its own promoter. Indeed, it appears that, in the well-studied cases, the auto-activation is typically mediated by a single (usually dimeric) binding site; examples include BaeR, CusR, PhoB, PhoP and ZraR. This is compatible with our predictions, because a dimeric binding site should yield a Hill coefficient between 1 and 2, depending on the dimerization kinetics. Note that the RRs that *inhibit* their own expression have more binding sites.

**Table 1:** Auto-regulating response regulators in *E. coli*.

Name	Sign	Evidence	Nr. binding sites	Source
ArcA	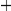	*	?	[47]
DpiA	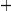	**	?	[48]
BaeR	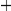	**	1	[35]
CusR	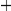	**	1	[49]
EvgA	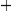	*	1	[50], [51]
KdpE	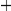	*	1	[52]
PhoB	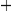	**	1	[34]
PhoP	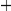	**	1	[32]
ZraR	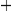	**	1	[37]
CpxR	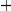	**	1–2	[53], [54]
QseB	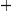	**	2	[55]
GlnG/NtrC	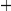 / 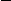	**	6	[56]
NarL	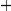 / 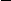	*	9	[57]
TorR	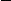	**	4	[58]

About half of the response regulators (RRs) in *E. coli* are known to auto-regulate; these are included in the table. (RRs that are not known to auto-regulate are: Atoc, BasR, CreB, DcuR, GlrR, NarP, OmpR, RcsB, RstA, UhpA, UvrY, YedW.) The second column of the table indicates the sign of the auto-regulation: + for activation, − for repression, +/− for both. Note, however, that not all TCS systems are well characterized. When the auto-regulation is inferred from expression studies only, so that indirect regulation is not obviously excluded, the column “evidence” contains a single star (*). If the RR has been shown to bind physically to its *cis*-regulatory region (by gel retardation studies with purified proteins or by footprinting essays), two stars are assigned. The column “nr. binding sites” specifies the number of binding sites found for the RR at its own promoter. Again, these data ought to be interpreted with care because, for instance, a single binding site for a RR acting as a tetramer can be hard to distinguish from two binding sites of a RR acting as a dimer. The table shows that, for RRs, auto-activation is more common than auto-repression, and that it is typically mediated by 1 or 2 binding sites.

Our other prediction is that the maximal fold-change of the auto-activating signaling circuits should not be high. Unfortunately, the fold changes of most TCS systems are not accurately known. One problem is that the native signals of many TCS systems are unknown. In addition, auto-activation may only be observed at a high signal level [30]. Also, if the introduction of the signal affects bacterial growth, global physiological effects have to be accounted for [31]. A subtle point is that the fold change 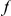, defined in terms of the regulatory function 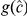, is generally not equal to the relative change in the steady state expression levels at 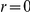 and 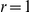, because even at 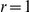 the circuit generally does not operate at the maximal transcription level 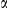. We can therefore provide only rough estimates. For PhoP a modest 10-fold increase in expression is reported between stimulated and unstimulated conditions [32]. Between phosphate-rich and phosphate-poor conditions, PhoB changes 12-fold according to Ref. [33], and 40-fold according to Ref. [34]. BaeR regulates its own operon, mdtABCD-baeSR, but the effect of BaeR amplification on baeS is only 10-fold [35], [36]; in addition, the putative inducer indole increases BaeR expression only 1.6 fold [36]. ZraR expression increases “significantly” in the presence of 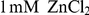, but a quantitative measurement of the fold change is, to our knowledge, not available [37]. In all these cases the basal expression is non-negligible and the fold change seems to be low to moderate, as expected from our analysis.

Few experiments have directly measured the induction time of signaling systems. An exception is the PhoP/PhoQ two-component system of *Salmonella* [38]–[40]. The PhoP response regulator auto-activates by binding to a single dimeric binding site. After induction, the mRNA level of PhoP needs 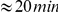 to reach its maximum value, which is about 20–30-fold higher than its basal level (depending on the signal); however, it takes about 30–40 min for the concentration of the protein PhoP to reach 50% of its maximum value [40]. These numbers agree well with our prediction in Fig. 5.

Induction times have also been measured for an entirely different class of auto-activating circuits: the type II restriction-modification (R-M) systems. R-M systems function as a defense against bacteriophages and are pervasive in bacteria; several thousands of putative R-M systems have been found [41]. These plasmid-borne systems consist of a restriction endonuclease (REase) that specifically cleaves DNA, and a methyltransferase (MTase) that methylates the same sequence and thereby protects it against the REase. Some R-M systems contain a third gene, called the C gene, which codes for a TF. C is co-transcribed with the REase, and in many examples regulates its own expression. For example, in the PvuII system, C binds to two dimeric binding sites, 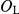 and 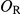, from which it respectively activates and represses its own expression [42]–[44]. The repressor site is much weaker than the activator site; it becomes relevant only at high C concentrations [44]. The auto-activation is believed to be important for the horizontal transfer of the plasmid between bacteria [42], [45]. Upon entering a cell, it is crucial that the MTase is expressed before the REase, to prevent the REase from damaging the new host's DNA. Indeed, auto-activation by C can provide such a delay. Experiments show that the induction of the C and REase proteins after entering the cell takes about 30 min [43]–a modest 10 min longer than the constitutively expressed MTase–and that the fold-change of auto-activation is ≳ 25 [43]. Despite the fact that a short delay is actually beneficial in this system, these numbers are compatible with the predictions in Fig. 5. The basal expression that is required to control the delay is provided by a separate, constitutive promoter [45], which suggest that natural selection has favored a short and predictable delay.

How can a cell reduce the induction time of an auto-activation circuit? Obviously, for a fixed fold change, the induction time can be decreased by using a high maximum transcription rate 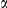. However, this would also result in a high expression level at full activation. Using typical numbers for *E. coli*: at a burst size 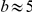 and a degradation/dilution rate of 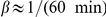, a high transcription rate 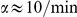 would lead to a steady state TF copy number of 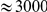, which is exceptionally high for a TF. To compensate, a low burst size (weak ribosomal binding site) could be used, and/or active degradation of the TF. Another option is to cap the maximal expression level by implementing auto-*repression* at high TF concentrations, on top of auto-activation at lower TF levels [46] (as is often found in type II restriction-modification systems, discussed above [42]). However, because auto-repression reduces the steady state expression at full induction, it also limits the effective fold-change achieved. Each of the above measures obviously introduces additional overhead, the cost of which should be weighted against a higher basal transcription level or a slower induction.

Finally, our analysis provides several testable predictions. In particular, the strong dependence of the induction time on the basal expression level could be tested experimentally in modified versions of the PvuII circuit, or in a synthetic system, similar to the one presented in Ref. [27]. One way to vary the basal expression level of a synthetic auto-activation circuit is to express the TF from two independent promoters, one of which can be controlled. By tuning the expression from this promoter, its impact on the induction time can be studied. We hope that our analysis inspires such experiments to characterize the importance of stochasticity in constraining the design of signaling circuits.

## Supporting Information

Text S1 **Methods, derivations and added analyses.** This document provides (i) detailed derivations of the mathematical results used in the main text, (ii) a description of the methods used to calculate mean induction times, and (iii) a discussion of the full induction time distributions. (Includes 6 figures.)
(PDF)
