# Supplementary material for: Speed, Sensitivity, and Bistability in Auto-activating Signaling Circuits
Source: PLoS Comput Biol. 2011 Nov 17;7(11):e1002265. doi: 10.1371/journal.pcbi.1002265 (PMC3219618; doi:10.1371/journal.pcbi.1002265)
Supplement: Text S1 — Methods, derivations and added analyses. This document provides (i) detailed derivations of the mathematical results used in the main text, (ii) a description of the methods used to calculate mean induction times, and (iii) a discussion of the full induction time distributions. (Includes 6 figures.) (PDF) [file pcbi.1002265.s001.pdf]

Supporting Text S1, for

SPEED, SENSITIVITY, AND BISTABILITY IN INDUCIBLE AUTO-ACTIVATION CIRCUITS

Rutger Hermsen*, David W. Erickson, Terence Hwa
Center for Theoretical Biological Physics and Department of Physics,
University of California, La Jolla, CA 92093-0374
* hermsen@ctbp.ucsd.edu

Contents

1 Shape of the response function 1

2 Calculation of the sensitivity 2
- 2.1 *Definitions* . . . 2
- 2.2 *Sensitivity of the open-loop circuit* . . . 2
- 2.3 *Sensitivity of the auto-activation circuit* . . . 3
- 2.4 *Threshold response conserved in the stochastic model* . . . 3

3 Fast calculation of the mean induction time 3
- 3.1 *Numerical first passage time calculation* . . . 4
- 3.2 *Application to the induction time* . . . 4

4 Induction time distributions 5

5 Bimodality vs. bistability 5
- 5.1 *Conditions for bimodality at $H = 1$ and $b = 1$.* . . . 5
- 5.2 *Parameter regions of bistability and bimodality* . . . 6

1 Shape of the response function

In the deterministic model presented in the main text, the dynamics of the TF concentration c was described by the following ordinary differential equation:

$$\frac{\mathrm{d}c}{\mathrm{d}t} = g(rc)(b/V) - \beta c, \tag{1}$$

in which b is the burst size, V the volume of the cell, and r the fraction of TFs that is modified/activated. The transcription rate $g(\hat{c})$, with $\hat{c} \equiv rc$, is affected by auto-regulation and is assumed to have the following form:

$$g(\hat{c}) \equiv \alpha \frac{(\hat{c}/K)^H + 1/f}{(\hat{c}/K)^H + 1}.$$

Here α is the maximal transcription rate, K is the dissociation constant of the TF binding to its binding sites, H is the Hill coefficient, and f the fold change.

The response function was defined as the steady state concentration of modified/activated TF as a function of r, denoted $\hat{c}_\mathrm{s}(r)$. Here we demonstrate that the *shape* of the response function is determined only by f and H. With this we mean that the other parameters merely scale the response function horizontally and/or vertically. Since scaling a function should not change its sensitivity, nor whether or not it has a bistable domain, we focus on the shape parameters f and H.

From Eq. 1, the steady state TF concentration c_s is implicitly given by

$$c_\mathrm{s} = [b/(\beta V)]\, g(rc_\mathrm{s}).$$

In terms of $\hat{c}_\mathrm{s} = rc_\mathrm{s}$, this can be written as

$$\hat{c}_\mathrm{s} = r[b/(\beta V)]\, g(\hat{c}_\mathrm{s}).$$

We now rewrite this equation in terms of the scaled and dimensionless quantities $\tilde{c} \equiv \hat{c}_\mathrm{s}/K$ and $\tilde{r} \equiv (c_\mathrm{max}/K)\, r$, where $c_\mathrm{max} \equiv (\alpha/\beta)(b/V)$:

$$\tilde{c} = \tilde{r}\, \tilde{g}(\tilde{c}). \tag{2}$$

Here $\tilde{g}(\tilde{c})$ is defined as

$$\tilde{g}(\tilde{c}) \equiv \frac{(\tilde{c})^H + 1/f}{(\tilde{c})^H + 1}. \tag{3}$$

Only two parameters remain: H and f. This shows that the other parameters merely scale the response function horizontally and/or vertically; therefore, the shape of the response function is determined by H and f only.

The case $H = 1$ (non-cooperative auto-activation) is of special interest, and can easily be solved exactly. In this case,

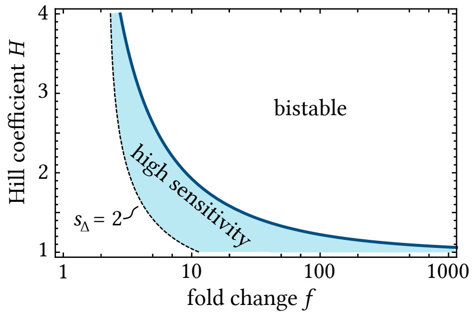

Figure S1: Phase diagram showing the sensitive regions of parameter space, according to the measure s_Δ. (In the main text a similar figure is provided for measure s_d.) Here, we have chosen a rather stringent cutoff of $s_\Delta = 2$, which compares to Hill functions with a Hill coefficient larger than 4. We note that, unlike s_d, s_Δ does depend on the threshold location $r_\mathrm{t} = K/c_\mathrm{max}$; we have used $r_\mathrm{t} = 0.5$ to generate this plot. Higher values of r_t lead to higher values of s_Δ for given f and H.

Eq. 2 is quadratic and yields:

$$\tilde{c}(\tilde{r}) = \frac{\tilde{r}-1}{2} + \frac{1}{2}\sqrt{(\tilde{r}-1)^2 + 4\tilde{r}/f}.$$

In the limit of large f, this reduces to $\tilde{c} = \tilde{r} - 1$, and $\tilde{c} = 0$ becomes a stable solution for $\tilde{r} < 1$, so that

$$\hat{c}_\mathrm{s}(r) = \begin{cases} 0 & \text{if } r \leq r_\mathrm{t}, \\ c_\mathrm{max} r - K & \text{if } r > r_\mathrm{t}. \end{cases} \tag{4}$$

(Here we defined $r_\mathrm{t} \equiv K/c_\mathrm{max}$.) This proves that in this limit the response becomes threshold-linear. Lastly, from Eq. 4, $c_\mathrm{s}(r)$ follows directly, yielding

$$c_\mathrm{s}(r) = \begin{cases} 0 & \text{if } r \leq r_\mathrm{t}, \\ c_\mathrm{max} - K/r & \text{if } r > r_\mathrm{t}. \end{cases}$$

2 Calculation of the sensitivity

The main text contains results about the sensitivity of the response function as a function of parameters. Here we derive these and additional results.

2.1 *Definitions*

We use two different measures to quantify the sensitivity of response functions $y(x)$. The first measure, $s_\mathrm{d}[y(x)]$, is defined as the maximum slope of $y(x)$ in a log–log plot:

$$s_\mathrm{d}[y(x)] \equiv \max_{x \in \mathbb{R}^+} \left(\frac{\mathrm{dlog}\, y(x)}{\mathrm{dlog}\, x} \right).$$

This measure is commonly used and effectively detects regions of large amplification or gain. However, a high s_d does not establish that $y(x)$ resembles a binary switch (or step function). Therefore we also introduce the measure s_Δ as

$$s_\Delta[y(x)] \equiv \frac{\Delta \log y(x)}{\Delta \log x},$$

where the Δ refers to the x-interval in which $y(x) - y(0)$ increases from 10% to 90% of its value. Thus, s_Δ can be interpreted as the average log–log derivative (also called gain) calculated over the domain in which the function switches from low to high. A high value of s_Δ therefore does indicate switch-like behavior.

2.2 *Sensitivity of the open-loop circuit*

In the open-loop circuit, the response is determined by the properties of the promoters of the genes regulated by the TF (its target genes). We assume that the expression of these genes responds according to a Hill function:

$$\tilde{h}(r) \equiv \alpha \frac{(r c_\mathrm{s}/K)^H + 1/f}{(r c_\mathrm{s}/K)^H + 1}.$$

Here c_s is the steady state TF level, and α, H, f and K are respectively the maximal transcription rate, the Hill coefficient, the fold change and the dissociation constant of the target promoter. As before, r is the fraction of the TFs that is activated. Because scaling of r and vertical scaling of $h(r)$ do not influence s_Δ, we continue our calculations in terms of the scaled Hill function $h(x)$, defined by

$$h(x) \equiv \frac{x^H + 1/f}{x^H + 1}.$$

The log–log derivative $d(x)$ of $h(x)$ can be calculated directly:

$$d(x) \equiv \frac{x}{h(x)} \frac{\mathrm{d}h(x)}{\mathrm{d}x} = \frac{(f-1)\, H\, x^H}{(1+x^H)\,(1+f x^H)}.$$

Maximizing this function gives

$$s_\mathrm{d}[h(x)] = \frac{H}{H_\mathrm{c}(f)},$$

where we defined

$$H_\mathrm{c}(f) \equiv \frac{\sqrt{f}+1}{\sqrt{f}-1}. \tag{5}$$

This result is used in the main text.

To calculate s_Δ for the same function, we first derive the value of x where $h(x) - h(0)$ reaches a fraction ϕ of its maximum value $1 - 1/f$, by solving

$$h(x) - h(0) = \phi\,(1 - 1/f).$$

The solution is $x^H = \phi/(1-\phi)$. This means that the switching domain is bordered by x_l and x_h such that $x_\mathrm{l}^H = 0.1/(1-0.1) = 1/9$ and $x_\mathrm{h}^H = 0.9/(1-0.9) = 9$. The corresponding values of $h(x)$ are $h_\mathrm{l} = 0.1 + 0.9/f$ and $h_\mathrm{h} = 0.9 + 0.1/f$ respectively. Therefore,

$$\begin{aligned} \Delta \log r = \Delta \log x &= \log x_\mathrm{h} - \log x_\mathrm{l} \\ &= \frac{2}{H} \log 9, \end{aligned}$$

and

$$\Delta \log h(x) = \log\left(\frac{9+1/f}{1+9/f}\right).$$

This results in

$$s_\Delta[h(x)] = \frac{\Delta \log h(x)}{\Delta \log x} = W(f)\left(\frac{H}{2}\right), \quad (6)$$

where the prefactor

$$W(f) = \left(\frac{\log(9+1/f) - \log(1+9/f)}{\log 9}\right) \quad (7)$$

converges to 1 from below in the limit of high f. Eqs 6 and 7 prove that, for Hill functions, s_Δ can be at most $H/2$.

2.3 *Sensitivity of the auto-activation circuit*

We now calculate the sensitivity of the auto-activation circuit. The response of the circuit is determined by Eq. 2. We cannot solve this equation analytically for general parameters. However, it is straightforward to find the *inverse* of the response function, $\tilde{r}(\tilde{c})$:

$$\tilde{r}(\tilde{c}) = \frac{\tilde{c}}{\tilde{g}(\tilde{c})}. \quad (8)$$

We can use this inverse function to calculate $s_\mathrm{d}[\tilde{c}(\tilde{r})]$. The log–log derivative of the response function at a given output $\tilde{c}$ is given by

$$d(\tilde{c}) = \frac{\tilde{r}}{\tilde{c}}\frac{\mathrm{d}\tilde{c}}{\mathrm{d}\tilde{r}} = \frac{\tilde{r}(\tilde{c})}{\tilde{c}} \Bigg/ \frac{\mathrm{d}\tilde{r}(\tilde{c})}{\mathrm{d}\tilde{c}} = \left[1 + H\left(\frac{1}{f(\tilde{c})^H+1} - \frac{1}{(\tilde{c})^H+1}\right)\right]^{-1}. \quad (9)$$

This function is maximal when $\tilde{c} = f^{-1/(2H)}$. By inserting this into Eq. 8 we learn that this occurs when $\tilde{r} = f^{(H-1)/(2H)}$, and by inserting it in Eq. 9 we obtain the sensitivity

$$s_\mathrm{d} = \frac{H_\mathrm{c}(f)}{H_\mathrm{c}(f) - H},$$

which is the result used in the main text. ($H_\mathrm{c}(f)$ was defined in Eq. 5.) For a given f, s_d diverges when $H \to H_\mathrm{c}(f)$; this is because the circuit is bistable when $H > H_\mathrm{c}(f)$.

The values of s_Δ for general parameters have to be calculated numerically. Fig. S1 shows the region of parameter space in which the response is sensitive, taking $s_\Delta = 2$ as the cutoff. (To obtain these results, we assumed that $K/c_\mathrm{max} = 0.5$.) We already showed that for Hill functions s_Δ is at most $H/2$, which implies that the sensitivity of auto-activating circuits with $s_\Delta > 2$ is similar to the sensitivity of Hill functions with $H > 4$. We conclude that, even though there are clearly numerical differences between s_d and s_Δ for the auto-activation circuit, the region of high sensitivity is similar for both measures.

One case of special interest can be worked out by hand: the non-cooperative auto-activation circuit ($H = 1$), in the limit of large f. We showed that, in this limit, the response becomes threshold-linear. The value of s_Δ now depends only on the location of the threshold, $r_\mathrm{t} \equiv K/c_\mathrm{max}$, and can be calculated straightforwardly, yielding:

$$s_\Delta = W(1/r_\mathrm{t})^{-1},$$

where W was defined in Eq. 7. For example, at $r_\mathrm{t} = ½$, this evaluates to $s_\Delta = 4.0$. To obtain the same value with a Hill function, the Hill coefficient $H = 8$ is required, which shows that the threshold response is highly sensitive.

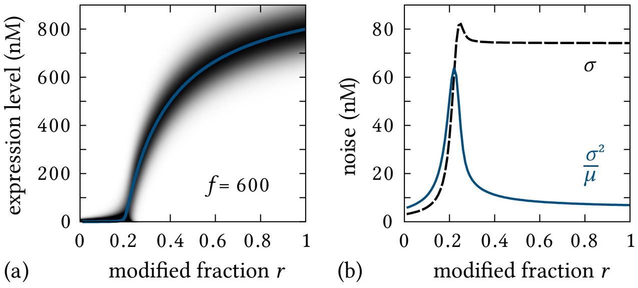

Figure S2: Response characteristics of the stochastic model at $H = 1$, at a high fold-change f. (a) Shown as a density plot is the steady-state probability distribution of the total TF expression level $c(r)$ as a function of the modified fraction r. For comparison the blue line shows the prediction from the deterministic model. As in the deterministic model, the stochastic model predicts a threshold response. (b) Shown are both the standard deviation σ and the Fano factor σ^2/μ (where μ is the mean) of the response plot in (a), as a function of r. The peak of the Fano factor in the threshold region is mainly caused by the reduced mean: σ peaks only mildly. Parameters: $\alpha = 4/\mathrm{min}$, $\beta = 0.04/\mathrm{min}$, $K = 200\,\mathrm{nM}$, $b = 10$, $V = (1\,\mu\mathrm{m})^3$, and $f = 600$.

2.4 *Threshold response conserved in the stochastic model*

The above calculations for the deterministic model demonstrate that, if $H = 1$ and f is large, an ultra-sensitive threshold response is obtained. Fig. S2 shows that this feature is conserved in the stochastic model. (A figure very similar to Fig. S2(b) is presented in Ref. [1] for a related model.)

3 Fast calculation of the mean induction time

In the main text, we discuss the induction time of the stochastic circuit. To calculate this quantity, we used the following fast method for calculating first passage times, which can be applied to a large class of models. We first describe the method in a general form, and then explain how we applied it to the calculation of the mean induction time.

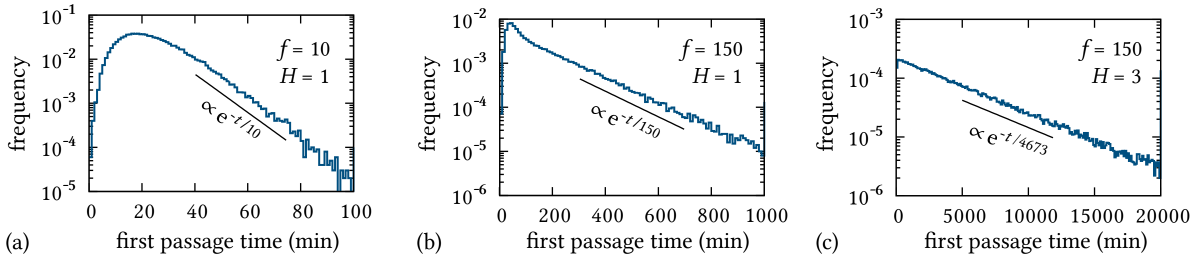

Figure S3: Induction time distributions, obtained by simulations (10^5 repetitions per histogram). In all three plots $K = 50/V$, $\alpha = 1$/min, $\beta = 0.04$/min and $b = 10$. (a) Results for $f = 10$ and $H = 1$. At these parameters, $f < \alpha/\beta$, so that the deterministic model and the mean of the stochastic model give similar results. (b) Distribution for $f = 150$ and $H = 1$. Now, $f > \alpha/\beta$; that is, anomalous induction occurs, and the induction is limited by the first transcription event, at rate $\alpha/f = (150\,\text{min})^{-1}$. As a result, the distribution is approximately exponential. (c) At $f = 150$ and $H = 3$ the model is in the classical bistable regime. As expected for waiting times of barrier crossing problems, the distribution is close to exponential.

3.1 *Numerical first passage time calculation*

We imagine a stochastic system with a countable number of states n. We ask how long it takes (on average) before a "random walker" exits the domain $n \in [0, a\rangle$ if at time $t = 0$ it is in state n with probability $p_{\text{in}}(n)$.

First, we write down a Master equation for the domain $n \in [0, a\rangle$ with an absorbing boundary condition at $n = a$. In vector notation, it reads

$$\frac{\mathrm{d}\vec{p}(t)}{\mathrm{d}t} = A\,\vec{p}(t),$$

where $\vec{p}(t)$ is a a-dimensional vector containing the probabilities $p_n(t)$ of being in state n at time t, and A is the $a \times a$ transition matrix. Given an initial condition $\vec{p}(0) = \vec{p}_{\text{in}}$, this linear system of differential equations has the formal solution

$$\vec{p}(t) = \mathrm{e}^{At}\,\vec{p}_{\text{in}}. \tag{10}$$

Since a is an absorbing boundary, the sum $S(t) \equiv \sum_{n=0}^{a-1} p_n(t)$ decreases with time; in fact, $S(t)$ is the probability that at time t the walker has not left the domain $n \in [0, a\rangle$ yet (*i.e.*, $S(t)$ is the survival probability). Let $F(t)$ denote the probability density that walker exits the domain for the first time at time t (*i.e.*, the first passage time probability density); then

$$F(t) = -\frac{\mathrm{d}S(t)}{\mathrm{d}t}.$$

Partial integration now shows that the *mean* first arrival time equals

$$\begin{aligned}\tau &= \int_0^\infty tF(t)\,\mathrm{d}t \\ &= \int_0^\infty S(t)\,\mathrm{d}t.\end{aligned} \tag{11}$$

(Here we assumed that $\lim_{t\to\infty} S(t) = 0$, that is, that the walker will surely be absorbed if we wait long enough.)

For brevity, we below use the notation $\sum \vec{v}$ to denote the sum of the elements of a vector $\vec{v}$. Then, from the Eq. 11, the definition of $S(t)$, and Eq. 10, it follows that

$$\begin{aligned}\tau &= \int_0^\infty \sum \vec{p}(t)\,\mathrm{d}t \\ &= \sum \int_0^\infty \mathrm{e}^{At}\,\vec{p}_{\text{in}}\,\mathrm{d}t \\ &= -\sum A^{-1}\,\vec{p}_{\text{in}}.\end{aligned} \tag{12}$$

This result shows that, in order to calculate the mean first passage time, one only needs to invert the matrix A, multiply it by the initial condition $\vec{p}_{\text{in}}$, and sum the elements of the resulting vector.

It remains to be shown that inverse A^{-1} indeed exists. For this, a sufficient condition is that $\lim_{t\to\infty} S(t) = 0$: this implies that all probabilities p_n must vanish with time, which means that all eigenvalues of A are negative. Then, the determinant of A is nonzero, A is non-singular, and the inverse A^{-1} exists.

In software packages such as Matlab, large, sparse matrices like A can be inverted quickly. But since we do not need the inverse matrix itself—just its product with $\vec{p}_{\text{in}}$—a fast way to implement Eq. 12 in Matlab is:

```
t=-sum(A\p).
```

where `p` is the initial condition $\vec{p}_{\text{in}}$. After defining `A` and `p`, the whole mean first passage time calculation can thus be implemented in a single line of code.

3.2 *Application to the induction time*

We define the induction time as follows. We imagine that the signal has been absent long enough to ensure that the circuit is in the steady state corresponding to $r = 0$. Then, at time $t = 0$, the signal is introduced ($r = 1$). The induction time is the mean time it takes before the TF expression level reaches 50% of the average steady state expression level at $r = 1$. Therefore, the induction time can be calculated using the method described above.

A subtlety is that, at time $t = 0$, the circuit can *already* be in a state $n \geq a$ (due to a fluctuation). This can easily be taken into account. We can write the mean first arrival time as

$$\tau = \sum_{n=0}^{\infty} p_{\mathrm{s}}(n)\, \tau_n,$$

where $p_{\mathrm{s}}(n)$ is the steady state probability distribution at $r = 0$, and τ_n is the mean first passage time given that, at time $t = 0$, the system is in state n. Now, if $n \geq a$ at $t = 0$, the induction time is zero, *i.e.*, $\tau_n = 0$. This means that we can truncate the summation at $n = a - 1$:

$$\tau = \sum_{n=0}^{a-1} p_{\mathrm{s}}(n)\, \tau_n.$$

Therefore we calculate $p_{\mathrm{s}}(n)$, and simply truncate it at $n = a - 1$; the resulting (unnormalized) distribution is then used as $\vec{p}_{\mathrm{in}}$ in the method explained above.

4 Induction time distributions

We defined the induction time as the *mean* time it takes, after the signal arrives, until the expression reaches 50% of the average steady state level. To verify that the mean time is representative of the underlying induction time probability distribution, we also studied the full distributions.

Fig. S3 shows distributions for three parameter settings, obtained by simulations of the induction process. The parameters in Fig. S3(a) are chosen such that $f < \alpha/\beta$, so that no anomalous induction is expected. The exponential tail of the distribution is determined by the time scale of the slowest step in the induction process, which is the transcription in the "off" state, *i.e.* $f/\alpha = 10\,\mathrm{min}$. Despite this exponential tail, the coefficient of variation c_{v}, defined as the standard deviation over the mean, is 0.50 for these parameters, showing that the distribution is more compact than exponential distributions. In additon, if both α and K are increased in proportion while other parameters are kept fixed, the process becomes more and more deterministic and c_{v} is further reduced (data not shown). In Fig. S3(b), $f > \alpha/\beta$ and $H = 1$, so that anomalous induction occurs. As we explain in the main text, now the induction time is dominated by the waiting time for the first transcription event. Because this is approximately a Poisson process, this leads to an approximately exponential induction time distribution, with scale parameter $f/\alpha = 150\,\mathrm{min}$. Consistently, the coefficient of variation for this distribution equals $c_{\mathrm{v}} = 0.96$, close to the value $c_{\mathrm{v}} = 1$ for exponential distributions. Lastly, Fig. S3(c) shows results for $f = 150$ and $H = 3$, which is in the deterministically bistable regime. Now, the induction process is a classical barrier crossing problem, and again an approximately exponential distribution should be expected [2]. Indeed, this is what we find, with $c_{\mathrm{v}} = 0.99$. Fig. S4 plots c_{v} for various f and H, which supports this picture.

We conclude that in all regimes the mean waiting time is a reasonable measure of the induction speed, but the variation in the waiting time can be large. This shows that at large f, the induction tends to become slow as well as unpredictable.

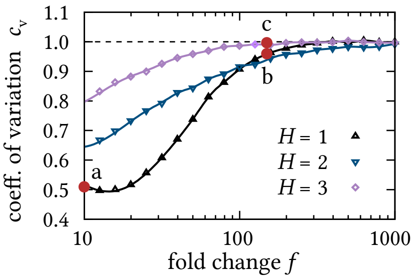

Figure S4: Coefficient of variation for the induction time distributions. The coefficient of variation c_{v} is defined as the standard deviation divided by the mean. The figure presents data obtained by simulation (10^5 repeats per data point; the errors are comparable to the symbol size). Results are shown for a range of f values, and for $H = 1, 2, 3$. The other parameters are as in Fig. S3. Independent of H, c_{v} converges to 1 for high fold changes; indeed, there the distributions are approximately exponential. Red dots and labels indicate the results corresponding to the distributions in Figs. S3(a), (b), and (c).

5 Bimodality vs. bistability

To be precise, we make a distinction between bimodality and bistability. A circuit is called bistable if the deterministic model predicts two stable steady states. We call it bimodal if the steady state probability distribution of the TF expression level, according to the stochastic model, has two peaks. If a circuit is bistable, one would expect it to be bimodal, and *vice versa*—but this is not always true [1, 3–8].

5.1 *Conditions for bimodality at $H = 1$ and $b = 1$.*

A non-cooperative auto-activation circuit ($H = 1$) cannot be bistable. Nevertheless, for certain parameter values, the stochastic model predicts that it is bimodal, even if the production of proteins is not "bursty" (burst size $b = 1$). This is demonstrated in Fig. S5. Here we derive the exact conditions for this to occur, because, as we argued, the anomalous induction time and anomalous bimodality are two sides of the same coin.

We first consider the ratio $R(n)$, defined as

$$R(n) \equiv \frac{p_{\mathrm{s}}(n+1)}{p_{\mathrm{s}}(n)},$$

where $p_{\mathrm{s}}(n)$ is the steady probability that n TFs are present in the cell. Because $b = 1$, detailed balance holds; for the Master equation given in the main text, this implies that

$$g(rn)p_{\mathrm{s}}(n) = \beta(n+1)p_{\mathrm{s}}(n+1),$$

so that

$$R(n) = \frac{g(r\,n)}{\beta(n+1)}.$$

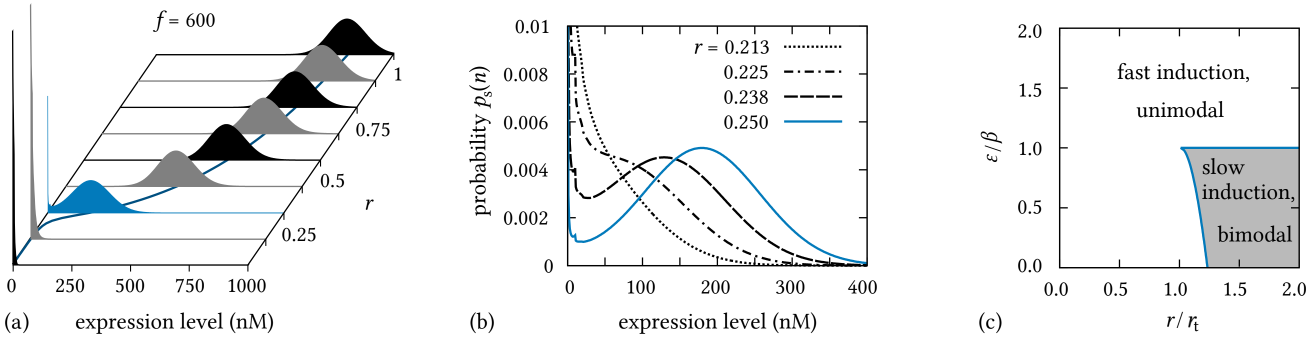

Figure S5: Anomalous bimodality. At $H = 1$ (non-cooperative auto-activation), even though the circuit is not bistable according to the deterministic model, bimodal expression is observed in the stochastic model if the fold-change f is high. (a) Steady-state distributions of the total TF concentration for various values of the modified fraction r, at $f = 600$. The blue line indicates the mean of the distributions as a function of r. Because $f > \alpha/\beta$ bimodality results: at $r > 0.23$ an unexpected peak appears at $n = 0$. This is particularly clear at $r = 0.25$ (shown in cyan). (b) Transition from unimodal to bimodal. If $r < 0.23$ the distributions are unimodal with a peak at $n = 0$; if $r > 0.23$ a second peak appears. If r is further increased, the peak at $n = 0$ shrinks, but technically never completely disappears. (c) Phase diagram summarizing the conditions for anomalous slowdown and bimodality in the stochastic model: the basal transcription rate $\epsilon \equiv \alpha/f$ must be lower than the protein degradation rate β, and the modified fraction r has to be sufficiently higher than the threshold $r_t \equiv K/c_{max}$ (see Eq. 17). Parameters (unless specified): $\alpha = 4$/min, $\beta = 0.04$/min, $K = 200$ nM, $V = (1\,\mu\text{m})^3$, $f = 600$ and $b = 10$.

In order to have bimodality with a peak at $n = 0$, there should be values n_1 and n_2 (with $n_1 < n_2$) such that $R(n) < 1$ if $0 \le n < n_1$ or $n > n_2$, and $R(n) > 1$ for values $n_1 < n < n_2$. In that case, n_1 can be identified as the minimum between the two peaks of $p_s(n)$, and n_2 marks the location of the second peak.

The condition $R(n) < 1$ is equivalent to the condition $Q(n) < 0$, where $Q(n)$ is the quadratic polynomial

$$Q(n) \equiv An^2 + Bn + C, \tag{13}$$

with

$$\begin{aligned} A &\equiv -\rho, \\ B &\equiv (\mu - 1)\rho - 1, \\ C &\equiv \mu/f - 1. \end{aligned}$$

Here we used the abbreviations $\mu \equiv \alpha/\beta$ and $\rho = r/K$. This means that bimodality occurs if and only if $Q(n)$ has two positive roots. The following three requirements are necessary and sufficient to ensure this:

1. $Q(0) < 0$; this immediately leads to the condition

$$f > \mu. \tag{14}$$

2. $-B/(2A) > 0$, because the top of the parabola $Q(n)$ (see Eq. 13) must occur at a positive n. Because $A < 0$ this requires $B > 0$, leading to

$$\mu > 1, \tag{15}$$

$$\rho > 1/(\mu - 1). \tag{16}$$

3. The determinant $D \equiv B^2 - 4AC$ must be positive. D itself can be interpreted as a quadratic function of ρ:

$$D(\rho) = A'\rho^2 + B'\rho + C',$$

with

$$\begin{aligned} A' &= (\mu - 1)^2, \\ B' &= -4[1 - \mu/f] - 2(\mu - 1), \\ C' &= 1. \end{aligned}$$

The constraint in Eq. 15 ensures that $A' > 0$. Furthermore, it guarantees that $B' < 0$. These two facts together prove that the minimum of $D(\rho)$ occurs at a positive value of ρ, since $-B'/(2A') > 0$. Also, because $(B')^2 - 4A'C' > 0$ (again using Eq. 14 and 15), we know that $D(\rho) = 0$ has two roots, x_1 and x_2, and since $D(0) = 1 > 0$ both roots must be positive. Therefore, $D(\rho)$ is positive provided $\rho < x_1$ or $\rho > x_2$ (we assume without loss of generality that $x_1 < x_2$). However, since $x_1 < 1/(\mu - 1) < x_2$, Eq. 16 eliminates the range $\rho < x_1$. We calculate x_2 and finally arrive at the condition

$$\begin{aligned} 0 &< 1/\rho \\ &< \mu + 1 - 2\mu/f - 2\sqrt{\mu - \mu^2/f - \mu/f + (\mu/f)^2}. \end{aligned} \tag{17}$$

To summarize, we proved that bimodality occurs exactly if $f > \alpha/\beta$ (required to have a peak at $n = 0$), $\alpha/\beta > 1$, and Eq. 17 holds (required to have a second peak). Very similar results have been obtained for a different stochastic model, presented in the Supplementary Material of Ref. [8].

For general b it is less straightforward to give a full proof of the conditions of bimodality. Yet, a peak at $n = 0$ only requires that $p_s(n+1)/p_s(n) < 1$, which for any b is equivalent to $f > \alpha/\beta$. Therefore, at any $b \ge 1$, if $f > \alpha/\beta > 1$ bimodality should be expected for some range of r/K.

5.2 *Parameter regions of bistability and bimodality*

In the main text, we treated bistability in the context of the deterministic model. This is convenient, because in that

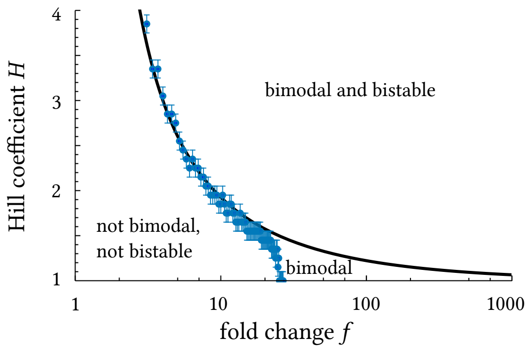

Figure S6: Bistability and bimodality compared. The black line $[H_c(f)]$ separates the region of parameter space where the circuit is monostable from the region where it is bistable (according to the deterministic model). The blue dots (with error bars) indicate where bimodality sets in according to the stochastic model. A rather high burst size $b = 10$ is chosen in order not to underestimate the effect of the noise. As long as $f < \alpha/\beta$, the bimodal and bistable regions coincide. At $f > \alpha/\beta$ and $H \approx 1$, the stochastic model is bimodal while the deterministic one is not bistable. Parameters used for the stochastic model: $\alpha = 1\,\mathrm{min}^{-1}$, $\beta = 1/25\,\mathrm{min}^{-1}$, $K = 50\,\mathrm{nM}$, $V = (1\,\mu\mathrm{m})^3$ and $b = 10$.

model the requirements for bistability can easily be calculated. Would our conclusions change if we, instead, would have used the stochastic model?

Fig. S6 shows in which domain of parameter space the system is bimodal (in the stochastic model); it also shows where it is bistable (in the deterministic model). Clearly, the two domains closely overlap, except in the region $H \approx 1$, $f > \alpha/\beta$, where the anomalous bimodality occurs that was discussed above. This means that requiring that the circuit is not bistable is practically the same as requiring that it is not bimodal. We are therefore justified to use the simple mathematical results from the deterministic model in the main text.

References

1. N. Friedman, L. Cai, and X. S. Xie, *Linking stochastic dynamics to population distribution: an analytical framework of gene expression*, Phys Rev Lett, 97 (2006), p. 168302.
2. C. W. Gardiner, *Handbook of stochastic methods for physics, chemistry, and the natural sciences*, Springer-Verlag, Berlin, 3rd ed ed., 2004.
3. A. Lipshtat, A. Loinger, N. Q. Balaban, and O. Biham, *Genetic toggle switch without cooperative binding.*, Phys Rev Lett, 96 (2006), p. 188101.
4. A. Ochab-Marcinek and M. Tabaka, *Bimodal gene expression in noncooperative regulatory systems*, Proc Natl Acad Sci U S A, 107 (2010), pp. 22096–101.
5. D. Schultz, J. N. Onuchic, and P. G. Wolynes, *Understanding stochastic simulations of the smallest genetic networks*, J Chem Phys, 126 (2007), p. 245102.
6. M. Scott, T. Hwa, and B. Ingalls, *Deterministic characterization of stochastic genetic circuits*, Proc Natl Acad Sci U S A, 104 (2007), pp. 7402–7.
7. M. Thattai and A. van Oudenaarden, *Intrinsic noise in gene regulatory networks*, Proc Natl Acad Sci U S A, 98 (2001), pp. 8614–9.
8. T.-L. To and N. Maheshri, *Noise can induce bimodality in positive transcriptional feedback loops without bistability*, Science, 327 (2010), pp. 1142–5.
